# A caring-perception model for ethical competence in virtual reality environment

**DOI:** 10.1177/09697330251328651

**Published:** 2025-03-19

**Authors:** Camilla Koskinen, Anne Gunn Dovland Vassbø, Linda Estman

**Affiliations:** 56627 University of Stavanger, Norway

**Keywords:** Qualitative research, empirical approaches, ethics of care/care ethics, theory/philosophical perspectives, moral sensitivity, professional ethics, ethics education

## Abstract

**Background:**

An ethical foundation for caring involves doing good, alleviating suffering, and treating human beings with dignity and respect. While virtual reality (VR) has primarily been used to develop clinical skills, there is limited research on its use for educating healthcare personnel in ethical competence and the use of VR grounded by ethical concepts and theories. This gap has prompted us to develop a theoretical basis grounded in enhancing the ethical competence of health professionals using VR.

**Aim:**

The study aimed to develop a caring-perception model for enhancing ethical competence in VR environments for educating healthcare personnel.

**Method and Material:**

The development of the caring-perception model was fundamentally anchored in theoretical frameworks established by the three caring theorists Eriksson, Martinsen, and Koskinen. Hermeneutic reading was used to interpret selected texts, extracting meaningful fragments to form interpretive patterns, leading to the creation of basic elements for a theoretical model. The caring-perception model was then interpreted in the context of developing ethical competence in a VR environment.

**Results:**

The caring-perception model consists of six fundamental elements: “I am here,” “I see and listen,” “I’m affected,” “I realize,” “I’m responsible,” and a synthesis in “ethical bearing and acting.” The theory model serves as a robust framework aimed at enhancing healthcare personnel’s ethical competence within VR environments.

**Discussion:**

VR grounded on a theoretical basis and educational model has the potential to offer unique opportunities to enhance healthcare personnel’s ethical competence and to practice ethical decision-making in simulated scenarios, fostering presence, attentiveness, and ethical reflection. Despite challenges such as technical barriers and the need for substantial investment, the potential benefits of using VR for ethical training can promise improved patient outcomes and a more ethically aware healthcare workforce.

## Introduction

This research is based on the premise that an ethical foundation for caring is to do good, alleviate suffering, and treat human beings with dignity and respect. According to Eriksson,^1^ ethics is in continuous motion, and when it serves as the foundation for caring, ethical values, compassion, and love emerge as natural acts of care in encounters with the suffering patient. Martinsen^2^ describes how professional judgment and understanding together with the senses, presence, and perception enable healthcare professionals to act and relate ethically toward the patient.

Ethical competence is a key concept in this study and is highlighted by Koskinen^3,4^ as a concept for ethical awareness, sensitivity, strength of character, moral judgment, responsibility, and willingness to do good. Birkelund^5^ attributes competence to qualities such as willingness to gain a deeper insight into and learn about life, being in harmony with life’s questions, and learning from other human beings. Competence, therefore, has an inherent potential and includes personal development and the ability to adapt to new situations and to act in new contexts.^6,7^ Competence is also dynamic; it is not a destination but a journey, and it is not just what one can do but also entails the ability to learn.^7,8^ According to Lind,^9^ ethical competence is not innate and does not develop on its own but must be learned, reinforced, and strengthened by exploring various methods that promote learning, reflection, and personal growth for empathy and awareness of others’ needs and feelings. Storaker et al.^10^ bring in an interesting dimension, namely, the importance of recognizing and taking care of one’s own vulnerability and of being affected by the patient’s vulnerability and how emotional immunization in turn can lead to moral blindness and make humans impervious to being emotionally affected. Moreover, Milliken and Grace^11^ emphasize the need for more research on methods for developing ethical awareness and sensitivity in encounters with the patient among healthcare professionals.

VR has primarily been used to develop clinical skills while our review of previous research shows that there is little research regarding its use as a method for the education of ethical competence among healthcare personnel^cf^^12^. This sparked our interest in developing a VR environment as an innovative method for enhancing ethical competence. Previous research also highlights the lack of a theoretical basis when educating the ethical competence of health professionals using VR. These introductory thoughts have challenged us to develop a theoretical basis grounded in caring theories for the use of VR to enhance health professionals’ ethical competence.

## Background

### Virtual reality in education and clinical practice

VR is a technology that creates environments which mimic human experiences through auditory, visual, and tactile feedback (including sight, sound, and touch), contributing to immersive and realistic scenarios.^13,14^ It allows users to interact with various dilemmas and scenarios in environments that are perceived as real, potentially enhancing a sense of presence. VR is used in education where students can practice different skills in various care environments, enabling more personalized education and a safe environment to learn and reflect, explore perspectives, and make risk-free decisions without exposing patients to harm.^15^ According to Wang et al.,^16^ VR provides the opportunity to experience a sense of presence and Vanlaere et al.^17^ highlight the relevance of VR for strengthening reflection in care practice and view vulnerability as a starting point for ethically responsible care. Technologies can be effective in enhancing empathy, presence, and immersion and in influencing the degree to which users can experience a sense of “being there” and how facial expressions can be immersive and evoke stronger emotional reactions.^14^

Studies^18–23^ show that VR increases engagement, improves learning outcomes, and promotes the development of critical thinking both in education and clinical practice within the healthcare sector. Research^16,19,24–27^ further reveals that the use of VR improves patient safety as training promotes communication, empathy, trust, and caregiving competence, which is the ability to understand others’ situations and to take care of others. According to Torda,^23^ VR that replicates real life makes it possible for users to fully focus on the scenario and stimulates both intellectual and emotional dimensions, thus contributing to the understanding of ethical issues that affect clinical decision-making. According to Wong et al.,^28^ the main advantages of virtual spaces are that they can make the invisible visible or at least highlight aspects that would otherwise receive little attention and alert caregivers to the most relevant issues when dealing with patients.

Despite the potential of virtual reality in education, there are ethical issues that must be considered. Fundamentally, scenarios in virtual reality should reflect real situations and portray people ethically and true to reality, without affecting the patient’s autonomy and the principle of not causing harm to others.^29^ The use of virtual reality can also induce psychological stress in participants, which should be carefully considered. High-flying virtual environments often provide diverse sensory impressions that can lead to overload, as they are directly connected to or near the participants’ senses.^30^ Additionally, there are recognized safety risks with virtual reality, such as motion sickness or “cyber sickness,” which can cause nausea, fatigue, dizziness, and eye strain.^14,22^

### The rationale for theory development

Integrating VR into education can promote a deeper understanding of ethical issues, strengthen the ability to make well-founded decisions, and prepare healthcare personnel for a complex reality in clinical practice. Rudschies and Schneiders’^22^ study shows that VR can evoke feelings of empathy, trust, and compassion in the same way as in genuine encounters. However, according to Salzmann-Erikson and Eriksson,^31^ and Fronczek,^32^ the transition to the digital age needs further research to avoid the neglect of a holistic human view and ensure that the use of VR is rooted in care and caring theories. Fronczek^32^ highlights the importance of a clearer and expanded theoretical focus in technology use, one that is anchored in nursing and caring theories and concepts to better capture the values of “being there” and “presence.” According to Francis,^33^ only scant research has focused on the importance of VR for ethical education, ethical competence, and decision-making. We have, therefore, considered it important to expand and anchor values of “being there” and “presence” to caring theories and ethical concepts as a theoretical basis when enhancing the ethical competence of health professionals’ education using VR.

## Aim

This study aims to develop a caring-perception model for education to enhance the ethical competence of health professionals using a VR environment.

## Method and Material

The research is methodologically grounded in Gadamer’s^34^ hermeneutic thinking. The pre-understanding is based on a review of previous studies, and the theoretical knowledge is rooted in the previous understanding of theories of caring and ethics within caring science. At the same time, the pre-understanding is put at stake to appropriate new understanding of the theories’ significance for enhancing ethical competence in a VR environment. The hermeneutic methodology involves a movement between reading, wonder, and interpretation of the selected theories, both as parts and as a comprehensive understanding.

According to Sanders et al.,^14^ VR technologies can increase empathy, presence and immersion, a sense of being there, and eliciting emotional responses. The inclusion criteria for the selection of theorists and texts were therefore that they should have a theoretical foundation in caring and ethics with a focus on “being there,” “presence,” “senses,” and “perception.” The material chosen for the study thereby consists of selected texts from Katie Eriksson, Kari Martinsen, and Camilla Koskinen (see Table 1).

**Table 1.** Material for the research.

Eriksson Katie	Jag var där, jag såg, jag vittnade och jag blev ansvarig – den vårdande etikens mantra. [I was there, I saw, I witnessed, and I became responsible – the mantra of caring ethics]. 2013
	Evidence. To see or not to see. 2010
	Evidens - det sanna, det sköna, det goda och det eviga. [Evidence - the True, the Beautiful, the Good and the Eternal]. 2009
	Mot en caritativ vårdetik. [Towards a charitable ethics of care]. 1995
Martinsen Kari	Disiplin og rommelighet. [Discipline and roominess]. 2003
	Øyet og kallet. [The Eye and the Call]. 2000
Koskinen Camilla	Lyssnande – ett vårdvetenskapligt grund- og praxisbegrepp. [Listening – a basic and practical concept in caring science]. 2022
	Lyssnande – en vårdvetenskaplig betraktelse. [Listening – a caring science contemplation]. 2011

**Figure 1.**
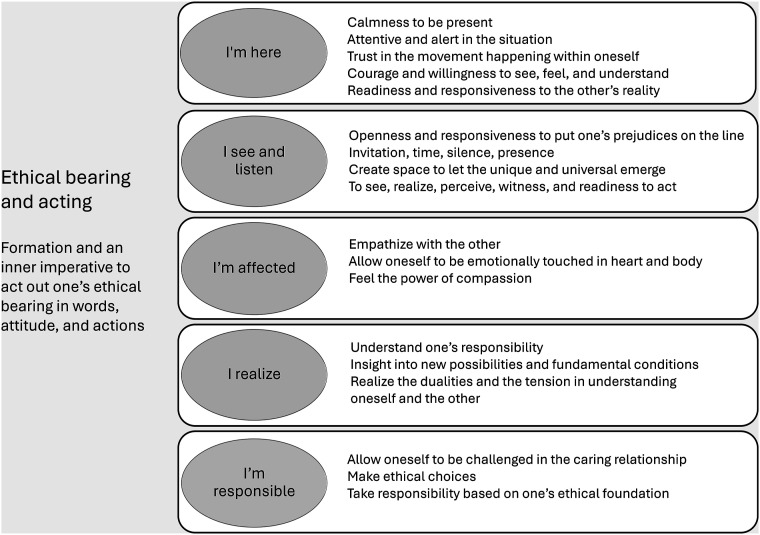
A caring-perception model for ethical competence education (CPECE model).

Hermeneutic reading according to Koskinen and Lindström^35^ was chosen as the method for reading and interpretation. First, the selected books and texts were read through to obtain an overall picture. Then, each work was read separately, and larger meaning-bearing sections were extracted for further reading. After a careful and thoughtful reading of the meaning-bearing text sections from the different texts, meaningful fragments were highlighted (see example in Table 2). Finally, the meaningful fragments formed interpretive patterns that were abstracted into basic elements for a theoretical model (see Figure 1). The creation of a theoretical model for education is an important tool that helps educators plan, implement, and evaluate education and learning in a structured way.

**Table 2.** Example of movement towards the creation of a theoretical model.

Meaningful fragments	Interpretive patterns	Basic elements for theory model
What manifests here and now (KE), attentive and alert (KM)	Calmness to be present	I’m here
Calmness and presence (KM), spaciousness, presence in time and space (KM) Trust that something is set in motion (KM)	Attentive and alert in the situation Trust in the movement happening within oneself	
Love for fellow humans (KM)	Courage and willingness to see, feel, and understand	
Dare to stand in the presence (KM), willingness to understand (KE)		
Not reducing the human (KE)		
At home in oneself in one’s own core values (CK)		
Mindset and attitude, courage and willingness to see, witness, love, and empathize with the patient (CK)	Readiness and responsiveness to the other’s reality	
See with one’s whole self and with a responsive gaze (KM)		
See reality with a sharp gaze (KE), listen to oneself to be able to listen to the other, existence/world (CK)

## Ethical reflections

Ethically, it has been important to be faithful to the theoretical substance and context of the selected texts. The theories and texts are not written with the context of VR in mind, and it has, therefore, been important to carry out the research in two stages, to first develop a general model for ethical competence, and then proceed to interpret and reflect on the model in relation to VR.

## Meaningful fragments

The results section begins with a description of meaningful fragments based on a hermeneutical reading of the three caring theorists’ texts.

### Meaningful fragments from Eriksson’s texts: The ethics of caring in the face of the suffering human being

According to Eriksson,^36^ technology has often been perceived as a threat to human values in human care, but there are no contradictions between charitable care and a high-tech care culture regarding the assumption that technology creates new opportunities for a richer existence for humans; nor does it reduce humans to a tool as long as the technology is based on ethical values and does not allow itself to be controlled by technological and economic interests. What is important is that the patient’s face is not hidden behind technology but that the face and the call of the face remain visible. Ethics becomes a reality only in moments where the other person’s face appears, and technology takes on a deeper meaning when the human face is seen, listened to, and allowed to emerge and awaken ethical responsibility. Hence, whether technology contributes to good care depends on how well health personnel master the art of caring itself. Ethics, seeing the patient, carrying responsibility, and having compassion, all become visible as a fundamental attitude, as an invitation, a presence, and being emotionally affected in a caring relationship. In encounters with the other, the healthcare personnel always have the greater responsibility.

Eriksson^37^ emphasizes that the words to see and to know have the same etymological origin in the Latin *video* which signifies “I see.” She also uses the term revision which means having a sharp eye to see reality for what it is and putting one’s own understanding and prejudices on the line. When seeing and gaining insight into different realities, pictures of the caring reality and a concrete situation can emerge. The word attest means to express and put into words what has been seen. Attestation, again, is an assessment of whether something is true. Thus, the person who has seen becomes a witness, a person who knows something and can make an attestation about something based on an extended understanding of what has been observed. Ethical testimonies correspond to the true, the good, and the beautiful.^38^ According to Eriksson,^37^ seeing and knowing involves a hermeneutic movement between understanding, interpretation, wholeness, and the unique and the special. For this movement to occur, the will to understand and absolute presence in the situation is necessary. Eriksson’s^38^ basic view of ethics leads to the importance of an inner value base or the human being who carries an ethos. Ethos becomes visible here and now as an ethical bearing. It is ethos that helps us to see and be responsive both to our innermost voice and to having the courage to meet the suffering human being in what Eriksson^39^ describes as the ethical mantra of care: I was there, I saw, I recognized, and I became responsible. It is thereby an ethical duty to be present, see, acknowledge, and take responsibility for providing good care to the suffering human being.

### Meaningful fragments from Martinsen’s texts: Seeing with the eye of the heart in patient encounters

Martinsen^40^ emphasizes the concept of seeing in a profound sense and uses the term “the eye of the heart,” in the sense of seeing with the eye of the heart, which is grounded in a heartfelt love for fellow human beings and the safeguarding and care of life. The eye of the heart has an active force and is a seat for emotions, thoughts, will, imagination, and compassion. The heart is the site of a human being’s ethical orientation and represents a call to compassion and effort; to perceive the other is already to be in an ethical relationship with the other. When one empathizes with the other, one is invited to follow along in the other’s world, meeting the other with a seeing-listening heart. The eye of the heart as seeing-listening also encompasses restraint so that the other can emerge in their integrity and vulnerable life context.^40^

Martinsen^40^ further describes the understanding of seeing-listening as a battle between seeing with a participating eye and seeing with a registrating eye. Ethics and professional judgment become central in this battle between seeing with the participating and registering eye. Martinsen^40^ also highlights the importance of seeing the face of the other. When we see the face of the other, we see the significance and vulnerability in every human being. We see the similarity, but also that the other emerges as themselves, in their difference. Seeing the face of the other is sensing and seeing with heartfelt, participatory eyes. Similarity and difference are not opposites, but fundamental conditions. Therefore, we must continually turn back to our everyday experiences and be attentive to what we have overlooked and already seen. When we see that another human being needs help, we must trust and allow ourselves to be set in motion. We must see with open eyes for it is the other who will emerge as significant and the one in need of help. We must let the other’s suffering touch our hearts and body. To see with the eye of the heart therefore means to see with one’s whole being and with a “listening gaze,” a gaze that is attentive and alert, characterized by calmness and presence, and that allows the ethical demand to become practical action and precede prejudices.^40^

Martinsen^2,40^ connects perception with professionalism. Senses and perception are open for seeing with emotions and when the suffering of the other matters, we obtain understanding. Seeing requires concentration in time and space, an intense presence with emotions, intellect, and the urge to act. This presence in time and space is called spaciousness; being touched and dynamically present, where time on the one hand is the societal clock time and on the other the time of life experience.^2^ Through dynamic and continuous self-formation, healthcare professionals can learn to know themselves and dare to stand in the presence of the other and not merely objectify; the evaluative and objectifying gaze of knowledge must be combined with being present, listening, and seeing. Furthermore, Martinsen^40^ uses the term slow eyes, in the sense of being participatory and attentively present, and combines this with professional observations and a “look for” gaze. Therefore, the eye of the heart is both perceptive and exploratory at the same time, evaluative and professionally open, and simultaneously open and unconditional, seeing the significance in all human life and allows it to be as it is. This openness to the diversity of experiences, where nurses listen to the tone of patients, forms the basis for knowledge acquisition. The inner ethics is about finding oneself together with others, being made responsible in community, in patient encounters.^2^

### Meaningful fragments from Koskinen’s texts: Listening as an ethical foundation in the encounter with the vulnerable human being

According to Koskinen,^41^ vulnerability and the ability to be affected by another human being are fundamental to caring. Therefore, it is important to dare to let oneself be affected and accept one’s own feelings and shortcomings. Koskinen’s^41^ clarification of the concept of listening shows that listening is connected with seeing, realizing, perceiving, observing, contemplating, and witnessing. The eyes and ears always intersect with, rely on, shape, and feed sounds and images to each other without excluding one or the other, while both are prepared for new understanding. Conceptually, listening can be understood as a mindset and a bearing, a willingness to dutifully see, witness, love, and empathize with the patient. According to Koskinen,^41,42^ listening is caring in itself. Every human being needs someone in their life to turn to, someone who will listen to their life story. People longing for someone who sees, takes time, is close, encourages, and supports the shaping of the spoken life narrative. Listening to life narratives requires time and consideration of one’s own feelings while receiving the narrative and making room for the dark and the light, both fear and hope. According to Koskinen,^42^ it is essential not to ignore the patient’s experiences and feelings, and to listen in silence, show availability and time, and openly receive the patient’s narrative in order to try to understand their suffering and vulnerability.

Koskinen^41,42^ describes how humans are inherently listening beings, and that listening is a human trait that encompasses listening to oneself, to others, and to existence or some form of higher power. Listening is also ethically grounded and is often seen as an ethical virtue and an ethos in terms of ethical choices and an ethical attitude toward self, the other, and the world. This ethical attitude is associated with silence, invitation, showing hospitality, and openness to the other’s otherness and vulnerability, feeling compassion for the other’s suffering. It is an insight into the other as otherness and as a mystery that calls for openness and making room for not knowing how the other feels or is. The other cannot be fully understood, but the other and the unique narrative can be opened and given meaning when the listener steps into the background. Listening thus involves being in this movement and tension between the unknown and the known. In such a listening encounter between individuals, it can feel as if time and space are erased, and we experience a sense of presence and participation in a larger shared narrative. Koskinen^42^ also underscores the importance of listening in relation to the other’s face, as it is the face that expresses suffering and asks and begs us not to be left alone. The eyes can plead urgently and call on our responsibility to see and listen.

Koskinen^42^ discusses listening in relation to the caregiver and practice. Listening requires self-critical reflection on one’s own interpretations and viewpoints to challenge one’s own understanding. Reflecting on one’s own practice necessitates a thinking through and willingness to understand rather than having a ready answer. Listening to others’ life narratives can therefore be described as a gift that can provide an opportunity to gain new insights, thereby growing as a person. To be able to invite the other into a relationship also involves being at home with oneself and one’s ethical core values. Listening is therefore an ethical choice and a responsibility, wonder, and also entails courage to be affected, especially when the patient’s narrative of vulnerability and suffering is experienced as challenging or threatening. Koskinen^42^ points out that it is only when the caregiver is touched by the other’s vulnerability and suffering that care can become caring.

### Creation of a caring-perception model for ethical competence education

Based on the understanding of the meaningful fragments from Eriksson (KE), Martinsen (KM), and Koskinen (CK), descriptive patterns were formulated and, finally, basic elements were abstracted for the creation of a theory model (see example in Table 2). Ultimately, a caring theory-based model for ethical competence was developed (see Figure 1) and interpreted in relation to enhancing ethical competence in a VR environment.

### Interpretation of the model for the development of ethical competence in a virtual reality environment

Ethical competence in healthcare professionals within virtual environments takes the form of a movement between “I am here,” “I see and listen,” “I’m affected,” “I realize,” “I’m responsible” to a synthesis in “ethical bearing and acting.”

#### I am here

VR environments provide training in being present and attentive and riveting the gaze on what appears and becomes visible in the moment, to be here and now, to see the other and feel compassion for the other. A requisite for stepping into a VR environment and being here is to be at home with oneself and one’s basic values and thereby to find the courage, willingness, and trust to be moved in the moment and have readiness and sensitivity to meet the other’s’ reality and feel with the other.

#### I see and listen

To be in a VR environment requires time, silence, and presence, to give space and receive the narrative and to create space where the uniqueness of the other can emerge. To see and listen requires sensitivity and self-critical reflection on one’s own interpretations and an openness to put one’s own prejudices on the line, to see and listen beyond what one has already seen and what exists beyond what is directly visible in the moment. Seeing and listening are woven together in the VR environment and the listening eye, and the seeing-listening heart becomes sharpened to participate, register, and look after. Participation in a VR environment enables presence where time and space disappear, and a sense of belonging to a more comprehensive narrative. The VR environment helps users to focus their senses, prepares them to witness the other’s suffering, and arouses their desire and will to act authentically and in a caring way.

#### I’m affected

The VR environment can be an expression of suffering and meeting the face of the other that asks not to leave the other alone. In the face of the other lies a vulnerability that evokes feelings of compassion, and this emotional affectedness prompts a responsibility to invite and show hospitality and openness to the other’s otherness. In the VR environment, there is time and space to be emotionally affected in the heart and body, to feel the power in compassion and what it means to have willingness to act.

#### I realize

The gaze that meets the observer in a VR environment can plead and call the observer to realize one’s responsibility to see and listen without reducing the other and bespeaks the impossibility of fully know or understand the other. To realize the importance of being affected as a way to understand the other, and simultaneously that the other in some respects is always a stranger. In moments when the other’s suffering begins to matter, one can realize and reflect over the duality and the tension in understanding self and the other and thereby obtain new insights into the basic assumptions of the universal in caring.

#### I’m responsible

VR environments offer users the opportunity to challenge themselves and so to develop both personally and professionally, to find an ethical value base and make ethical choices. VR environments give time for responsibility to grow and finding new forms for the art of caring and for ethical and caring relationships.

#### Ethical bearing and acting

In the VR environment, one can see and witness what speaks to the heart to find one’s own value base and ethical bearing. VR environments can help users to discover the core in all care and caring, because in the heart resides the human ethical orientation that calls us to ethical acting and arouses a desire to protect life and take care of the other human being.

## Discussion and implications

The development of the caring-perception model for ethical competence (CPECE) in this study is fundamentally anchored in caring theories established by Eriksson, Martinsen, and Koskinen. Each of these theorists provides unique insights into the concepts of caring and ethics. By grounding our model in these theories, we have constructed a robust framework aimed at enhancing the education of ethical competence within VR environments. Lacasse et al.^43^ underscore the critical importance of employing theories and conceptual frameworks in health science education. They argue that such frameworks not only enhance the sustainability of educational programs but also make them more adaptable to various clinical contexts. This alignment with theoretical foundations ensures that healthcare education remains relevant and effective in preparing both healthcare students and personnel for the ethical challenges they face in their practice.

VR environments offer unique opportunities for developing ethical competence. They can simulate real-life scenarios and allow healthcare students and personnel to practice and refine their ethical decision-making skills in a controlled setting.^15^ The immersive nature of VR creates conditions conducive to presence, attentiveness, and ethical reflection, which are essential for ethical competence. In virtual environments, healthcare students and personnel can experience a sense of being “here and now,”^16^ and the ability to be fully present and attentive to the situation at hand allows students to perceive and respond to ethical issues as they arise. This presence is not just physical but also involves a heightened state of awareness and focus on the ethical dimensions of care.

However, some researchers argue that the immersive nature of VR might not fully replicate the nuances of real-life interactions, which potentially limits the depth of ethical engagement.^44^ There is also a concern that the art and practice of care might lose the sense of presence and human connection in a virtual environment.^30^ Moreover, a potential challenge is that there may be technical and logistical barriers, such as the availability of VR equipment and the need for technical support.^13,14^ Integrating VR into education requires substantial investment in both time and resources, which may not be feasible for all institutions. Another potential challenge is the resistance to adopting new technologies, which can stem from a lack of familiarity with or skepticism about the effectiveness of VR training.^45^ Despite these challenges, the potential benefits of using VR to enhance ethical competence are substantial, which makes it a promising tool for the future of healthcare education. In turn, this can lead to improved patient outcomes and a more ethically aware healthcare workforce.^27^

The caring-perception model for ethical competence education (CPECE) can be practically tried out and applied in healthcare education and continuing education, particularly in VR environments considering that VR technology simulates realistic clinical situations, helping students and staff to enhance skills, especially given sense, presence, perception, and affectedness. Through the model, participants can reflect on their decisions and receive feedback and thus improving ethical reasoning. The model can also aid in developing ethical guidelines and policies within healthcare organizations. It can be used in research to evaluate educational methods and interventions, leading to better healthcare quality and patient outcomes. Grounded in the theories of Eriksson, Martinsen, and Koskinen, the model ensures relevance and adaptability in different clinical contexts. The model also has relevance to be tried out in other education areas. In education, students can work with the model before and after VR films and reflect on ethical elements. This approach supports the formation of ethical attitudes and actions, making the model essential in professional education. The next step is to try out the model in the education and training of health professionals and whether the model needs further development to become a valuable tool for enhancing ethical competence in various educational and clinical settings.

## Conclusion

A lack of a theoretical basis when educating and enhancing the ethical competence of health professionals using a VR environment challenged us to develop an educational model grounded in caring theories by Eriksson, Martinsen, and Koskinen. The starting point was the idea that VR offers unique opportunities for immersive and interactive learning experiences and that the selected literature had a theoretical foundation in caring and ethics with a focus on “being there,” “presence,” “senses,” and “perception.” Hermeneutic reading of selected theories resulted in The caring-perception (CPECE) model with six fundamental elements: “I am here,” “I see and listen,” “I’m affected,” “I realize,” “I’m responsible,” and a synthesis in “ethical bearing and acting”. The next step is to try out the model as a tool for awareness, reflection, and sensitive interactions in ethics education and training for nurses and other professionals. As a theoretical model, the caring-perception model is general enough to be tried in varied teaching methods and complex real-world healthcare settings and to determine whether the model needs further development to become valuable for enhancing health professionals’ ethical competence in various educational, clinical, and cultural settings.
